# Case report: Cardiac myxoid fibrosarcoma: a report of two cases

**DOI:** 10.3389/fonc.2025.1567625

**Published:** 2025-07-17

**Authors:** Weikai Dong, Zhaoqi Du, Dianxiao Liu, Lijuan Yang, Wei Li

**Affiliations:** ^1^ Department of Cardiovascular Surgery, Binzhou Medical University Hospital, Binzhou, Shandong, China; ^2^ Department of Medical Research Center, Binzhou Medical University Hospital, Binzhou, Shandong, China

**Keywords:** cardiac myxoid fibrosarcoma, diagnosis, surgery, treatment, prognosis

## Abstract

**Introduction:**

The research into cardiac tumors can be traced back to the 18th century, when Bonet first introduced the relevant concept. It was not until 1936 that the first successful resection of a cardiac tumor was performed. From a pathological origin perspective, cardiac tumors can be categorized into two main types: primary tumors, which originate from the heart itself, and secondary tumors, which result from metastases of malignant tumors in other tissues or organs. Primary cardiac tumors are exceedingly rare, among primary cardiac tumors, roughly 75% are benign, while the remainder are malignant. In contrast, approximately 75% of primary malignant cardiac tumors are sarcomas. Cardiac myxoid fibrosarcoma stands out as a particularly rare diagnosis in this domain.

**Case report:**

Case one: A 25-year-old man presented with chest pain and tightness. After initial treatment, his symptoms recurred and worsened. Imaging revealed a large mass in the left atrium obstructing the mitral valve. He underwent surgical resection of the tumor, thrombectomy, and tricuspid valvuloplasty. Pathology diagnosed myxoid fibrosarcoma. The patient was readmitted 7 months later with hemoptysis due to tumor recurrence and was lost to follow-up after symptomatic treatment. Case two: A 67-year-old woman was admitted with cough, chest tightness, and shortness of breath. Physical examination and imaging showed a mass in the left atrium causing mitral valve obstruction. She underwent surgical resection of the tumor. Pathology confirmed myxoid fibrosarcoma. After 6 months of follow-up, there was no tumor recurrence or metastasis.

**Discussion:**

Myxoid fibrosarcoma located in the left atrium can lead to mitral valve obstruction, causing symptoms of mitral stenosis such as dyspnea, cough, hemoptysis, and reduced exercise tolerance. Surgery remains the primary treatment for primary left atrial malignant tumors. Once the diagnosis is confirmed, active surgical intervention should be performed to relieve blood flow obstruction, remove pericardial effusion, and alleviate cardiac compression, which can extend patients’ lives in the short term and improve their quality of life. Despite advancements in diagnostic techniques and surgical methods, the prognosis for patients with cardiac tumors still depends on the histology and location of the tumor.

## Introduction

Cardiac myxoid fibrosarcoma is a rare and aggressive malignancy that typically originates in the left atrium. It is characterized by symptoms such as chest tightness, dyspnea, cough, and hemoptysis, often leading to mitral valve obstruction and mimicking mitral stenosis. The tumor’s rapid progression and serious consequences necessitate prompt diagnosis and treatment. Imaging modalities like echocardiography, CT, and MRI are crucial for visualizing the tumor’s size, location, and relationship with surrounding tissues. Pathological examination, including immunohistochemical staining for markers like Vimentin, SMA, and Desmin, confirms the diagnosis. Surgical resection is the primary treatment, aiming to remove the tumor, alleviate cardiac compression, and restore normal function. Despite advancements, the prognosis remains poor due to high recurrence and mortality rates. Long-term follow-up with regular imaging and laboratory tests is essential for monitoring recurrence and metastasis. Multidisciplinary collaboration is vital for managing this complex condition, and future research is needed to improve diagnostic and therapeutic strategies.

## Case one

A 25-year-old man presented with unexplained chest pain and tightness three months ago. His condition improved after receiving anti-infection treatment and closed thoracic drainage at another hospital. However, the chest tightness recurred, and he was admitted to the hospital one week ago due to a worsening of his original symptoms. The patient had no history of other diseases or treatments. Physical examination revealed coarse breath sounds in both lungs, with no dry or wet rales detected. His heart rate was 114 beats per minute, with a regular rhythm and hyperactive P2 tone. A grade III/VI diastolic murmur was heard in the mitral valve auscultation area, and there was no edema in either lower limb.

**Figure 1:**
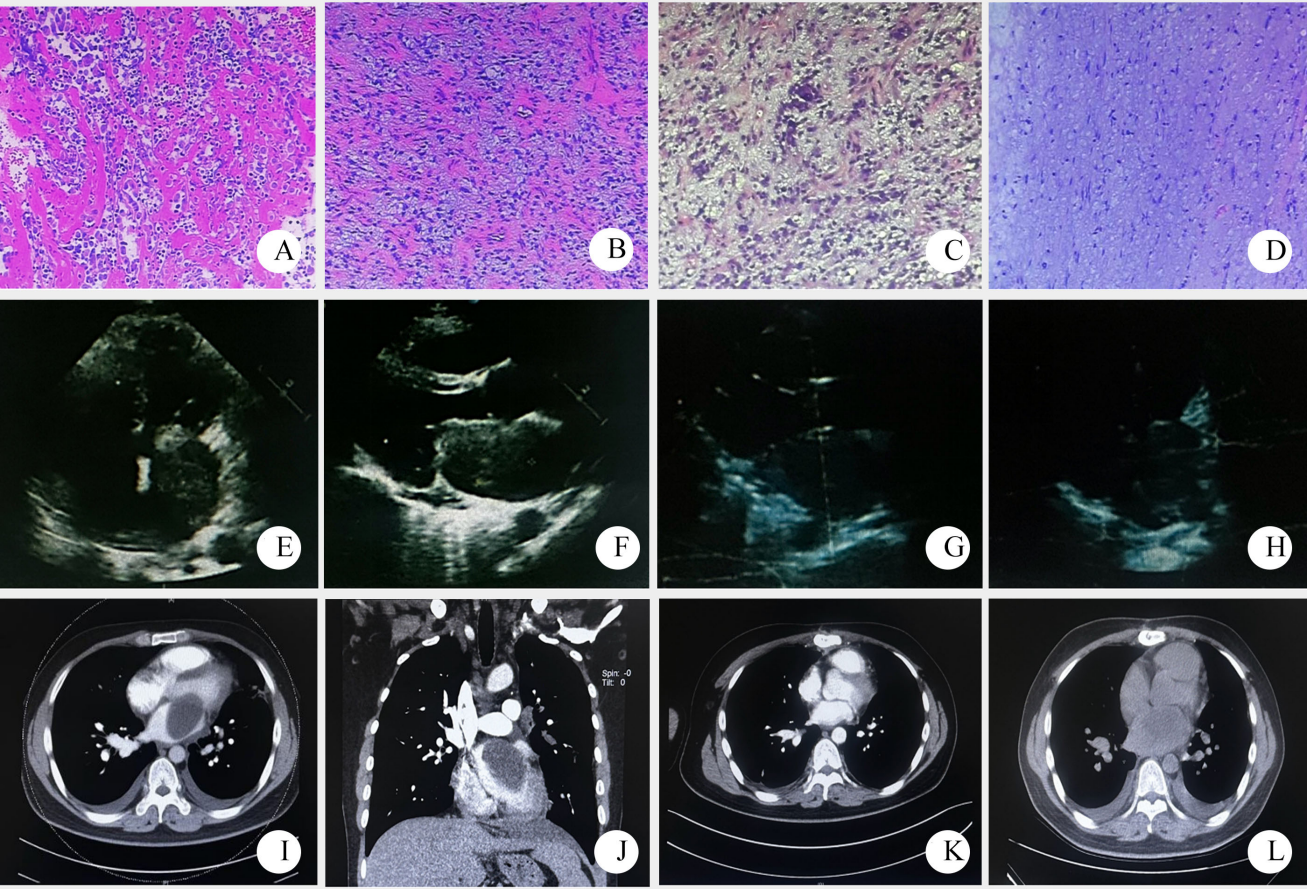
Pathological and imaging data of patient one (**(A–D)** pathological HE stained sections, 10×10; **(E, F)** a mass echo of 50mm×43mm×56mm was detected in the left atrium by echocardiography before operation. **(G, H)** postoperative cardiac color Doppler ultrasound; **(I, J)** preoperative pulmonary artery CTA showed large patchy low density shadow in the left atrium; **(K)** postoperative CTA; **(L)** 7 months later, chest CT showed low density shadow in the left atrium, considering recurrence).

**Figure 2:**
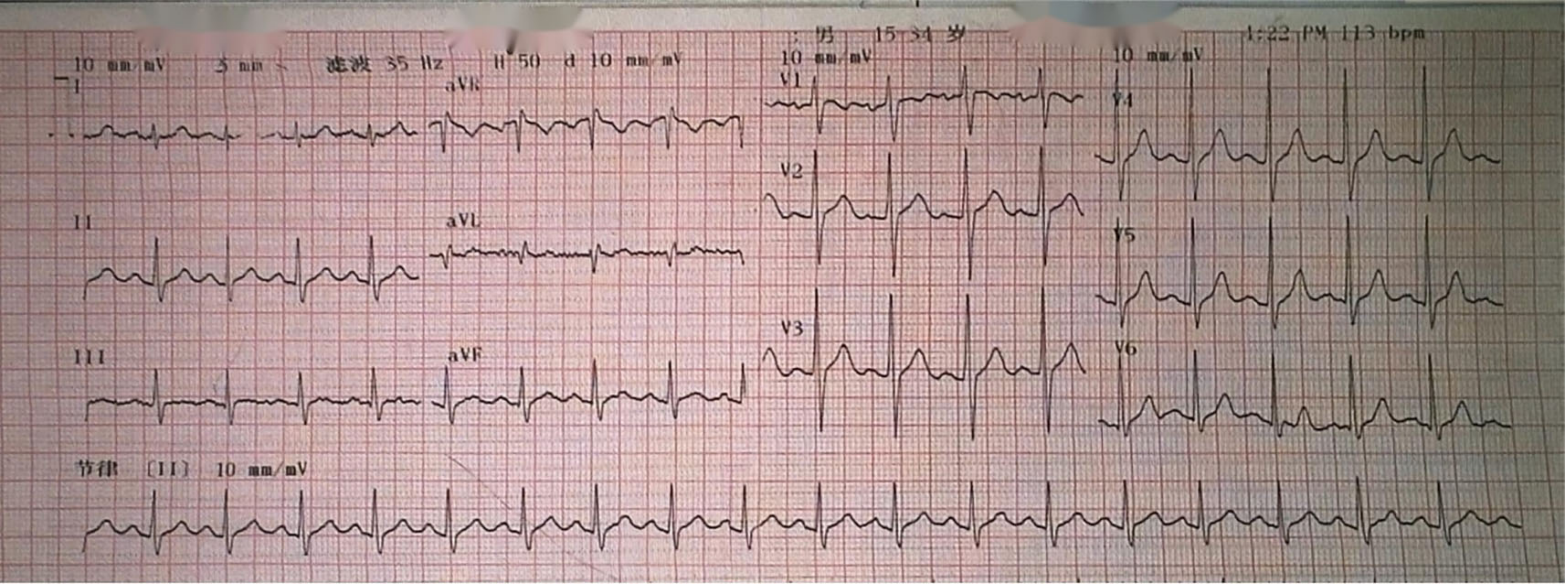
Patient one had sinus tachycardia on an ECG.

Auxiliary examinations revealed the following findings: A cardiac color Doppler ultrasound (**Figures 1E, F**) detected a mass in the left atrium measuring 50mm×43mm×56mm. The mass appeared relatively homogeneous with poor activity. Its pedicle seemed to be connected to the mitral valve, causing obstruction of the mitral valve orifice during diastole. A computed tomography angiography (CTA) of the pulmonary artery showed a large hypodense shadow in the left atrium, with a maximum diameter of approximately 54mm×51mm. Part of the shadow protruded into the left ventricle, and no enhancement was observed (**Figures 1I, J**). An electrocardiogram (ECG) revealed sinus tachycardia (**Figure 2**). Upon admission, the following laboratory findings were noted: creatine kinase isoenzyme was 13.4 U/L, creatine kinase was 39.3 U/L, NT-proBNP was 927.70 pg/mL, alkaline phosphatase was 126.7 U/L, γ-glutamyl transpeptidase was 55.6 U/L, and C-reactive protein was 43.10 mg/L. All other indicators were within the normal range.

Surgical procedure: The patient underwent left atrial tumor resection, pulmonary vein thrombectomy, and tricuspid valvuloplasty (**Figures 1G, H, K**). Upon opening the pericardium, external cardiac exploration revealed significant dilation of the right atrium and ventricle. The ascending aorta measured approximately 3.5 cm in diameter, and the main pulmonary artery measured about 3.0 cm in diameter, with no other external cardiac malformations observed. Generalized hepatic congestion was noted. Extracorporeal circulation was established, and 1000 mL of HTK cardioplegic solution was infused. The pericardial cavity was packed with ice slush. The right atrium and interatrial septum were incised, revealing a mass within the left atrium, measuring approximately 8 cm × 6 cm × 5 cm. The mass was solid, firm in texture, encapsulated, with a broad base, infiltrating the superior and lateral walls of the left atrium and completely obstructing the orifice of the left upper pulmonary vein. Numerous satellite nodules of varying sizes, ranging from 0.5 cm to 1.5 cm in diameter, were observed around the tumor, with some involvement of the right upper pulmonary vein orifice. Intraoperatively, the tumor was suspected to be malignant, with a high likelihood of sarcoma. The majority of the tumor was resected at its base, and the resulting defect was treated with electrocautery. Thrombus formation was detected within the left upper pulmonary vein and was thoroughly removed. The mitral valve was tested for competence and found to be satisfactory without intervention. Moderate tricuspid regurgitation was noted, and the tricuspid valve was repaired by folding the posterior leaflet to create a bicuspid configuration, with satisfactory results. The interatrial septum was closed with 3–0 Prolene suture, and the right atrial incision was closed with 5–0 Prolene suture. The superior and inferior vena cava were unclamped, the patient was rewarmed, and the left heart was vented. The ascending aorta was then unclamped. The heart spontaneously resumed beating with ventricular fibrillation, which was successfully converted to sinus rhythm with a single 20-joule defibrillation. Circulatory support was continued until hemodynamic stability was achieved. All cardiac cannulas were sequentially removed, and protamine was administered to neutralize heparin. The patient was on bypass for 132 minutes, with aortic cross-clamp time of 68 minutes. Urine output during the procedure was 200 mL, and ultrafiltration volume was 4500 mL, with no hemoglobinuria observed. Postoperatively, the patient was safely transferred to the intensive care unit with an initial blood pressure of 103/36 mmHg, heart rate of 120 beats per minute, and oxygen saturation of 100%.

Pathological diagnosis: The tumor was identified as a left atrial sarcoma. Immunohistochemical results revealed positive staining for Vimentin, weakly positive for SMA, and positive in a few cells for Desmin. S-100, CD31, and CD34 were all negative. The Ki-67 proliferation index was approximately 10%, leading to a diagnosis of myxoid fibrosarcoma (**Figures 1A–D**).

Prognosis: The patient was readmitted to the hospital 7 months after surgery due to hemoptysis. Imaging suggested tumor recurrence (**Figure 1L**). The symptoms were slightly relieved after symptomatic and supportive treatment. The patient was lost to follow-up after discharge.

## Case two

A 67-year-old female patient was admitted to the hospital due to a 1-month history of cough and expectoration, and 15 days of chest tightness and shortness of breath. The patient was unable to lie flat and had reduced exercise tolerance. Physical examination revealed weak breath sounds in both lungs, with dry and wet rales heard. There was no precordial bulge, the apex beat was normally positioned, no pericardial friction was noted, no cardiac thrill was palpable, and the cardiac dullness was normal on percussion. The heart rate was 106 beats per minute, with a regular rhythm, hyperactive P2, a diastolic murmur heard in the mitral valve area, and moderate pitting edema in both lower limbs.

**Figure 3:**
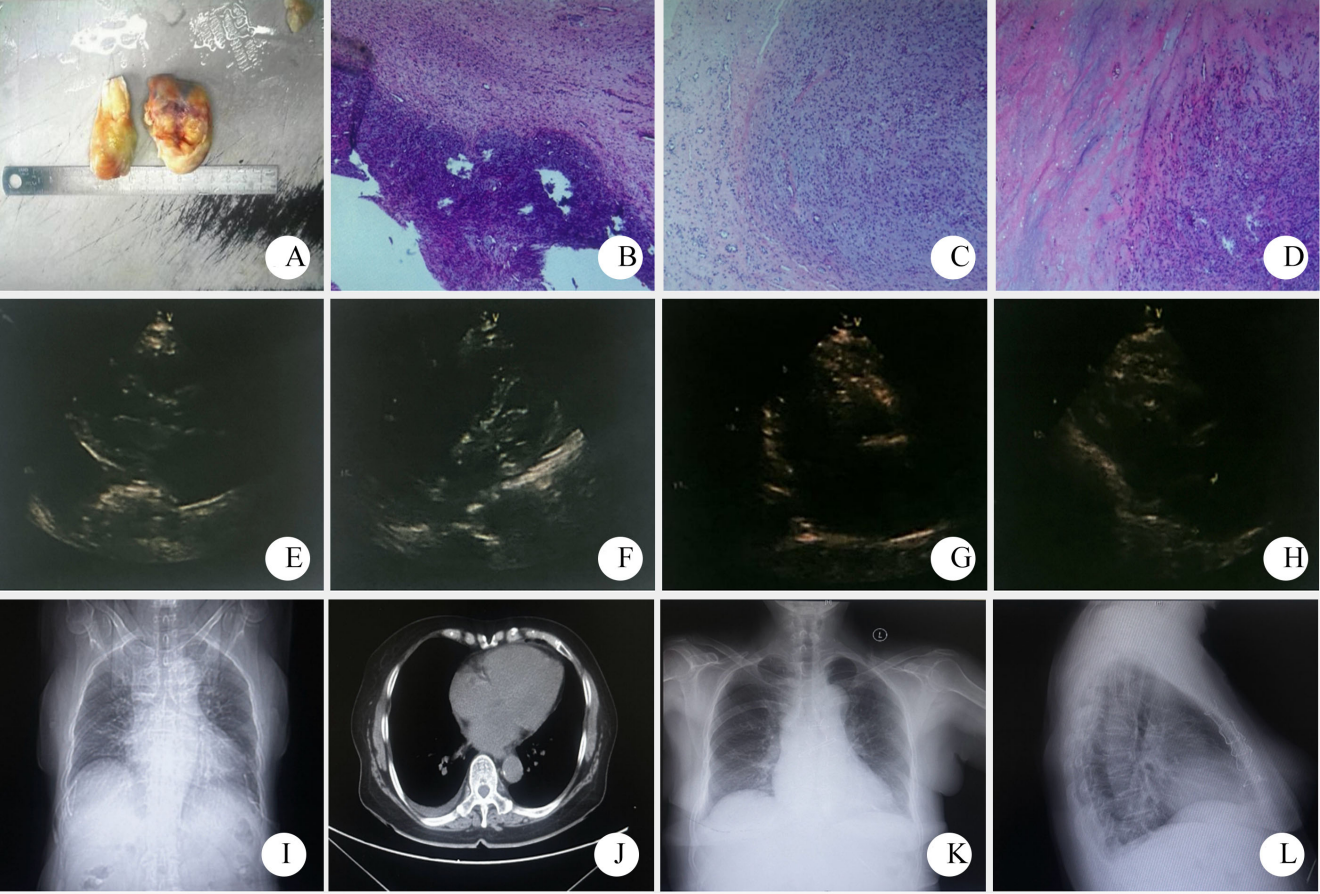
Pathological and imaging data of patient two (**(A–D)** gross observation and HE stained sections of the tumor, 4×10, 10×10, 10×10; **(E, F)** preoperative echocardiography showed a moderate to low echo of about 59mm×17mm in the left atrium. **(G–H)** postoperative cardiac color Doppler ultrasound; **(I, J)** Preoperative chest CT showed left atrial enlargement and right pleural effusion. **(K, L)** Chest X-ray reexamination at 6 months after surgery showed no obvious abnormalities after cardiac surgery).

**Figure 4:**
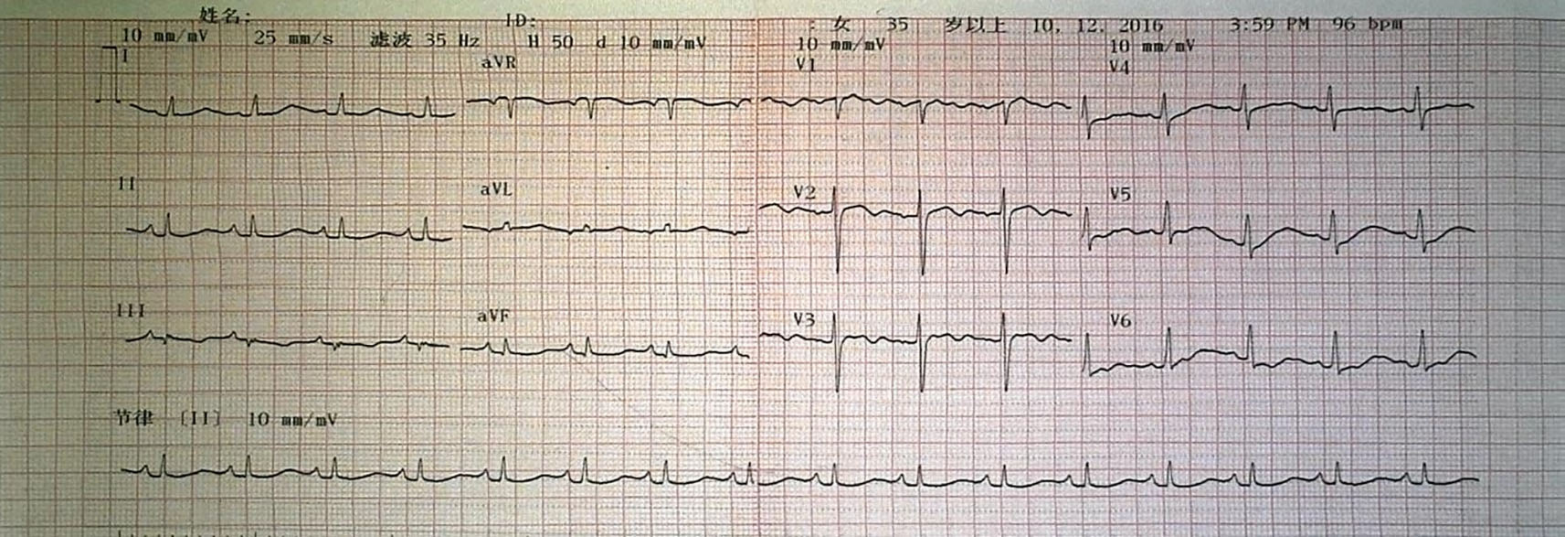
The ECG of patient two showed sinus rhythm with low T wave.

Auxiliary examination: Cardiac color doppler ultrasound (**Figures 3E, F**) revealed a moderately low echo mass in the left atrium, approximately 59mm×17mm in size, with a loose texture and connected to the atrial septum. During diastole, the mass protruded into the left ventricle, obstructing the mitral valve orifice, with an ejection fraction (EF) of 60%. Chest CT (**Figures 3I, J**) showed enlargement of the left atrium and right pleural effusion. An electrocardiogram (ECG) demonstrated sinus rhythm with low T waves (**Figure 4**). Upon admission, the following laboratory findings were noted: hemoglobin concentration was 101 g/L, hematocrit level was 33.8%, lymphocyte percentage was 14.0%, neutrophil percentage was 79.5%, fibrinogen concentration was 5.02 g/L, D-dimer level was 0.99 mg/L FEU, and total bilirubin was 27.40μmol/L. All other indicators were within the normal range.

Surgical procedure: The patient underwent left atrial tumor resection (**Figures 3G, H**). Upon opening the pericardium, external cardiac exploration revealed a mildly enlarged heart with edema of the epicardium and adventitia of the vessels. The ascending aorta measured approximately 4.0 cm in diameter, and the main pulmonary artery measured about 3.5 cm in diameter, with increased tension. Extracorporeal circulation was established, and 1000 mL of HTK cardioplegic solution was infused. The pericardial cavity was packed with ice slush for cooling. Access to the left atrium was achieved via a right atrial–septal incision, and a left heart decompression catheter was placed through the right upper pulmonary vein. Exploration revealed a tumor located in the left atrium, with its pedicle attached to the anterior margin of the foramen ovale. The main body of the tumor was resected along the pedicle. The tumor base measured approximately 3 cm in diameter, was bilobed, pale yellow, partially translucent with a jelly-like appearance, and had an intact capsule. After tumor removal, further exploration showed that the anterior, superior, and inferior margins of the foramen ovale were involved by the tumor pedicle. Residual tissue was carefully removed. The anterior and posterior leaflets of the mitral valve, as well as the anterior annulus, were densely adherent to the tumor. A 0.5 cm×1.0 cm tumor was present on the free edge of the anterior leaflet (A2 region), causing localized mitral valve insufficiency. The tumor and adherent tissue on the annulus and leaflets were meticulously removed, and the mitral valve was tested with water injection, showing no significant regurgitation. The left atrium and ventricle were thoroughly irrigated, and no residual tumor was found. Exploration via the right atrial incision revealed that the tricuspid annulus could accommodate three transverse fingers of the surgeon, and water injection testing showed no significant regurgitation. Rewarming was initiated, the interatrial septal incision was closed, the left heart was fully vented, and the ascending aorta was unclamped. The heart spontaneously resumed beating with sinus rhythm, a ventricular rate of 50 beats per minute, and multiple atrial premature contractions. An epicardial pacing lead was therefore sutured for temporary pacing. The right atrial incision was closed, the superior and inferior vena cava were unclamped, and circulatory support was continued until hemodynamic stability was achieved. The patient was gradually weaned from bypass, protamine was administered to neutralize heparin, and meticulous hemostasis was ensured. The patient was on bypass for 110 minutes, with aortic cross-clamp time of 50 minutes. Urine output during the procedure was 400 mL, and ultrafiltration volume was 2000 mL, with no hemoglobinuria observed. The patient was safely transferred to the intensive care unit postoperatively with an initial blood pressure of 101/64 mmHg, heart rate of 62 beats per minute, and oxygen saturation of 100%.

Pathological diagnosis: The tumor was identified as a left atrial sarcoma. Immunohistochemical results showed positive staining for Vimentin, weakly positive for SMA, and positive in some cells for Desmin. S-100 was negative, while CD31 and CD34 were positive in the vascular components. The Ki-67 proliferation index was approximately 30%, leading to a diagnosis of myxoid fibrosarcoma (**Figures 3A–D**).

Prognosis: The patient has been followed up for 6 months (**Figures 3K, L**), and no tumor recurrence or metastasis was observed during this period.

## Discussion

Cardiac tumors are a rare condition with an extremely low incidence rate, ranging from 0.0017% to 0.28% (1). Among these tumors, malignant ones account for 25%, with 95% being sarcomas and the remaining 5% consisting of lymphomas and mesotheliomas (2). Cardiac sarcomas make up 75% of malignant cardiac tumors and often present with atypical symptoms. The most common symptom is dyspnea, while others include chest pain, congestive heart failure, fever, general fatigue, and weight loss (3). Kim et al. (4) reported that cardiac sarcomas can be characterized on echocardiography by the following features: 1) The tumor does not occur in the septum. 2) The sarcoma can invade the pulmonary vein. 3) Multiple sarcomas. 4) Extensive attachment to the left atrial wall. 5) Tumor liquefaction can occur. Studies have found that CT and MRI enhancement is prominent and heterogeneous, with dynamic enhanced scans showing progressive enhancement. Primary cardiac fibrosarcoma is a malignant mesenchymal tumor, also known as myxofibrosarcoma due to its rich mucoid matrix. Primary myxofibrosarcoma is rarely found in the heart, accounting for only about 3% of cardiac malignant tumors and most often involving the left atrium (5). This tumor is distinguished from fibrosarcoma by the presence of giant cells and spindle or whorled cells (9). Currently, the pathogenesis of this tumor remains unclear. Some studies suggest that it originates from multipotential endothelial reserve cells (6), which is a type of myxoma with malignant transformation of latent malignant cells within the tumor during growth (7, 8). Myxoid fibrosarcoma located in the left atrium can cause mitral valve obstruction and cause symptoms of mitral stenosis, such as dyspnea, cough, hemoptysis, and decreased exercise tolerance (9). Surgical operation is still the main treatment (10, 11). After the diagnosis is confirmed, active surgical treatment should be performed to relieve blood flow obstruction, remove pericardial effusion and relieve the compression of the heart. Despite advances in diagnostic techniques and surgical approaches, this disease still has a poor prognosis.

The diagnosis of cardiac myxoid fibrosarcoma primarily relies on imaging and pathological examinations. Imaging modalities encompass echocardiography, CT, and MRI. Color doppler echocardiography can delineate the tumor’s size, shape, location, and activity, though early-stage tumors may be challenging to detect. CT and MRI offer clearer visualization of the tumor’s boundaries and its relationship with surrounding tissues, aiding in the assessment of tumor invasion and staging. Pathological examination stands as the gold standard for diagnosing cardiac myxoid fibrosarcoma. Tumor tissue, obtained via surgical resection or needle biopsy, undergoes histological and immunohistochemical analyses. Immunohistochemical examination identifies specific tumor cell markers, such as Vimentin, SMA, Desmin, etc., which assist in determining the tumor type and origin.

The two patients with cardiac myxoid fibrosarcoma reported in this paper presented with tumors located in the left atrium, symptoms of chest tightness, and P2 accentuation on auscultation. At initial diagnosis, both patients had heart rates exceeding 100 beats per minute, with electrocardiography showing sinus rhythm, indicative of sinus tachycardia. The maximum diameter of the tumors was greater than 5.0cm, and the tumors obstructed the mitral valve during diastole. There was no significant change in ejection fraction (EF). Chest CT revealed left atrial enlargement. The tumors were encapsulated and pedicled. During surgery, it was found that the tumors had infiltrated surrounding tissues, indicating strong invasiveness. The tumors were not easily detected by non-invasive examinations (such as cardiac color Doppler ultrasound) in the early stage. The histopathological features of the tumor were characterized by a heterogeneous cellular morphology, comprising spindle-shaped cells, round cells, and multinucleated giant cells, all embedded within a myxoid stromal matrix. Immunohistochemical staining revealed positive expression of vimentin, smooth muscle actin (SMA), and desmin, while S-100 protein was negative.

The primary treatment for cardiac myxoid fibrosarcoma is surgical resection, which aims to remove the tumor as completely as possible, alleviate the compression of the heart and great vessels, and restore normal cardiac function. Owing to the rarity of these tumors, surgical treatment is often referenced from the management of other types of cardiac sarcomas. Lars Niclauss et al. (12) retrospectively analyzed the clinical data of 9 patients with cardiac sarcoma and found that the 1-year mortality rate of cardiac sarcoma was 44%. However, surgery could significantly alleviate cardiac symptoms and improve survival rates. Complete tumor resection, absence of metastasis, and low-grade sarcoma types were associated with better prognosis. Rieneke Moeri-Schimmel et al. (13) reported two cases of primary cardiac sarcoma and reviewed the data of patients treated in three sarcoma centers in the Netherlands from 2005 to 2019. They concluded that surgery combined with postoperative radiotherapy was feasible for resectable cardiac sarcomas, although distant metastasis occurred frequently. For unresectable cardiac sarcomas, initial radiotherapy should be considered. Shusaku Maeda et al. (14) reported a case of a 19-year-old male patient with left ventricular synovial sarcoma. The patient presented with dyspnea and orthopnea. Imaging revealed a heterogeneous enhancing mass over 15 cm in the left ventricle, severely compressing the left atrium. Despite surgical resection and adjuvant chemotherapy and radiotherapy, the patient died of local recurrence 36 months postoperatively, indicating a poor prognosis for cardiac synovial sarcoma, especially in patients with extensive necrotic tissue. This is also similar to the treatment situation of case one in our study. A review by Pietro Scicchitano et al. (15) on Primary Soft Tissue Sarcoma of the Heart (pSTS-h) indicated that surgery is the primary treatment for pSTS-h, although chemotherapy and radiotherapy can also be effective in certain circumstances. All the above studies emphasize that surgery remains the mainstay of treatment for cardiac sarcomas. In addition, Hua Li (16) retrospectively analyzed 46 patients with non-metastatic primary cardiac sarcoma treated with heart transplantation and found that the median survival of heart transplant recipients was 16 months. Among them, the median survival of patients with angiosarcoma was 9 months, significantly lower than that of patients with other histological types (36 months). Low-grade and low-invasive histological subtypes benefited more from heart transplantation. Hassiba Smail et al. (17) reported a case of a patient who underwent total cardiac resection and total artificial heart implantation due to tumor invasion of the ventricle. They suggested that total artificial heart implantation is an effective treatment for unresectable cardiac tumors. These two studies indicate that heart transplantation can also be considered for patients with unresectable primary cardiac sarcoma.

Walid K. Abu Saleh et al. (18) retrospectively analyzed the clinical data of 44 patients with right-sided cardiac sarcoma from 1990 to 2015. Among them, 32 patients received neoadjuvant chemotherapy. The median survival of patients who received neoadjuvant chemotherapy was 20 months, significantly higher than that of patients who did not receive chemotherapy (9.5 months). The median survival of patients with complete resection (R0) was 53.5 months, significantly higher than that of patients with partial resection (R1) (9.5 months). They concluded that neoadjuvant chemotherapy combined with radical surgery could significantly improve the survival rate of patients with right-sided cardiac sarcoma. Gal Aviel et al. (19) introduced a novel treatment method—endovascular brachytherapy. They treated a 35-year-old male patient diagnosed with unresectable high-grade endocardial sarcoma involving the right ventricle and pulmonary artery. After three cycles of doxorubicin and ifosfamide treatment, the patient’s right heart failure symptoms worsened. Through a femoral vein approach, a brachytherapy catheter was placed in the right ventricle, main pulmonary artery, and right pulmonary artery, delivering a dose of 20 Gy for 10 minutes. Ten months later, imaging showed a significant reduction in tumor volume, an increase in the cross-sectional area of the pulmonary artery, a significant decrease in pulmonary artery pressure, and complete relief of heart failure symptoms. They concluded that endovascular brachytherapy is a new, safe, and effective treatment for unresectable primary cardiac sarcoma, which can be used to relieve obstruction or reduce tumor volume to achieve complete resection. These two studies provide valuable insights into the efficacy of chemotherapy. Anna Romanowska et al. (20) conducted intensity-modulated radiation therapy on two male patients with cardiac intimal sarcoma (CIS) and found that radiotherapy might be effective and tolerable in the treatment of CIS. However, given the risk of radiation-induced heart disease (RIHD) due to high-dose radiation directly targeting the heart, patients are expected to develop RIHD earlier than reported in the literature. Therefore, regular cardiovascular assessments are required.

Therefore, we believe that for patients with cardiac myxoid fibrosarcoma that have not developed distant metastasis, aggressive surgical treatment should be undertaken. During the surgical procedure, every effort should be made to achieve complete resection of the tumor tissue, thereby aiming for a radical cure and potentially delaying the risk of tumor recurrence or metastasis. For tumors that cannot be completely resected, palliative surgery may be considered to alleviate symptoms and improve quality of life. Moreover, adjuvant chemotherapy should be administered postoperatively, and radiotherapy may be employed when necessary. The primary chemotherapy regimen should be based on anthracycline agents. Additionally, the use of sensitizers (21, 22) or dendritic cell (DC) nanovaccines (23) may be considered as alternative therapeutic approaches.

Cardiac myxoid fibrosarcoma has a poor prognosis, characterized by high recurrence and mortality rates. Recurrent tumors tend to be more malignant and less responsive to treatment. Therefore, long-term postoperative follow-up with regular imaging and laboratory tests is essential to monitor for tumor recurrence and metastasis. Timely treatment should be administered if recurrence or metastasis is detected. Moreover, the patient’s psychological state and quality of life are crucial, necessitating psychological support and rehabilitation guidance to help patients better cope with the disease and treatment. Cardiac myxoid fibrosarcoma is a rare and highly malignant cardiac tumor, and its diagnosis and treatment require multidisciplinary collaboration. Early diagnosis, active surgical intervention, and comprehensive adjuvant therapy are key to improving patient survival rates and quality of life. Future research should focus on further investigating the pathogenesis and biological characteristics of cardiac myxoid fibrosarcoma, as well as exploring new diagnostic and therapeutic methods to enhance patient prognosis.

## Data Availability

The original contributions presented in the study are included in the article/**Supplementary Material**. Further inquiries can be directed to the corresponding author.
